# Innate Immune Responses in Leprosy

**DOI:** 10.3389/fimmu.2018.00518

**Published:** 2018-03-28

**Authors:** Roberta Olmo Pinheiro, Veronica Schmitz, Bruno Jorge de Andrade Silva, André Alves Dias, Beatriz Junqueira de Souza, Mayara Garcia de Mattos Barbosa, Danuza de Almeida Esquenazi, Maria Cristina Vidal Pessolani, Euzenir Nunes Sarno

**Affiliations:** ^1^Leprosy Laboratory, Oswaldo Cruz Institute, Oswaldo Cruz Foundation, Rio de Janeiro, Brazil; ^2^Cellular Microbiology Laboratory, Oswaldo Cruz Institute, Oswaldo Cruz Foundation, Rio de Janeiro, Brazil

**Keywords:** leprosy, innate immune responses, skin, *Mycobacterium leprae*, autophagy, toll-like receptors, inflammasomes

## Abstract

Leprosy is an infectious disease that may present different clinical forms depending on host immune response to *Mycobacterium leprae*. Several studies have clarified the role of various T cell populations in leprosy; however, recent evidences suggest that local innate immune mechanisms are key determinants in driving the disease to its different clinical manifestations. Leprosy is an ideal model to study the immunoregulatory role of innate immune molecules and its interaction with nervous system, which can affect homeostasis and contribute to the development of inflammatory episodes during the course of the disease. Macrophages, dendritic cells, neutrophils, and keratinocytes are the major cell populations studied and the comprehension of the complex networking created by cytokine release, lipid and iron metabolism, as well as antimicrobial effector pathways might provide data that will help in the development of new strategies for leprosy management.

## Transmission of Leprosy

Leprosy is a chronic granulomatous disease, which affects dermis and peripheral nerves and also can involve the eye, the mucosa of the upper respiratory tract, muscle, bone, and testes, caused by the intracellular pathogen *Mycobacterium leprae* (1, 2).

Early diagnosis of leprosy is a prerequisite for effective therapy and rehabilitation. According to Ridley (3) the earliest lesion in leprosy is an intraepidermal lymphocytic infiltration. Although the transmission pathways of *M. leprae* are not fully understood (4), there are several results that suggest that *M. leprae* is mainly dispersed by the nose, not the skin. The protective mucosal innate mechanism in the respiratory tract may contribute to low infectivity of *M. leprae* after exposition. The release of bacilli by multibacillary patients supports a respiratory transmission (5). Viable bacilli have been found for at least 2 days in discharged nasal secretion (6). The hypothesis of respiratory transmission is validated by studies that demonstrated that adhesins present in *M. leprae* surface, like heparin-binding hemagglutinin and histone-like protein may attach in alveolar and nasal epithelial cells and both cell types are capable of sustaining bacterial survival (7, 8). In addition, a previous study demonstrated that mce1a gene is found in *M. leprae* genome and that mce1a product is associated with *M. leprae* entry into respiratory epithelial cells (9).

## Histopathological Features in Leprosy

The association of the histopathologic aspects and the immune state of the patient has made it the basis of the all leprosy classification and has helped to understanding the immunologic background of this disease and its transmission.

The histopathology of the nose demonstrates that the majority of all bacilli are present mainly in macrophages, as observed in lepromatous skin and other tissues. Bacilli were also seen inside monocytes, Schwann cells, polymorphs and columnar and goblet cells of the pseudostratified epithelium, secretory gland, and ducts (10).

Ridley and Jopling (11) classification establishes that the disease may present different clinical forms that may vary accordingly to histopathological findings and the immune status of the host. Tuberculoid or paucibacillary leprosy is characterized by cell-mediated immune responses to mycobacterial antigens and low infection whereas lepromatous or multibacillary leprosy is characterized by humoral immune response and high bacillary load. The different degree of cellular immune response to *M. leprae* is responsible for different types of granulomatous reaction. Analysis of skin lesion cells demonstrated that epithelioid cells are usually seen in paucibacillary patients [tuberculoid (TT) and borderline tuberculoid], whereas foamy macrophages are found in multibacillary cases [borderline lepromatous (BL) and lepromatous lepromatous (LL)]. Macrophages may present a granular eosinophilic cytoplasm with large numbers of bacilli in early and active lesions. In older lesions, on the other hand, cells are highly vacuolated and the cytoplasm has a foamy appearance (1). Recent studies have demonstrated that the macrophages in lepromatous skin cells are positive for ADRP, suggesting that their foamy aspect may be derived from lipid bodies accumulation induced by *M. leprae* (12, 13).

Two types of leprosy reactions may occur in leprosy patients. Reversal reaction is an acute inflammatory episode in skin and nerves that occurs because of an increase or emergence of cellular immunity against *M. leprae* antigens in lower or previously non-responder patients and may occur in patients of the whole clinical spectrum, except the tuberculoid, TT form (14). In addition, neuritis is frequently associated with reversal reaction episodes. Erythema nodosum leprosum (ENL) occurs in approximately 50% of patients from lepromatous pole due to a complex interaction between innate and cellular immunity poorly understood. Reversal reaction lesions show activated epithelioid macrophages, organized or not as granuloma (15, 16). The hallmark of ENL is an infiltrate of neutrophils in the profound dermis and hypodermis, frequently accompanied by macrophages (17–20). However, neutrophils are not always present (21–23) and skin fragments collected after 72 h demonstrate the presence of lymphocytes, plasma cells, and mast cells (24).

The pathogenesis of nerve destruction varies accordingly the clinical form of the disease (25); although the understanding of mechanisms associated with nerve damage and regeneration in leprosy-associated neuropathy are not fully understood (26). In the pure neural leprosy, bacilli are rarely detected despite the clinical neurological impairment. In multibacillary cases, which show macrophages in considerable numbers within the nerve, bacilli are in greater numbers, often as large bundles or globi. Ultrastructural analyses demonstrate that BL and LL foamy macrophages and vacuolated Schwann cells contain numerous electrondense structures considered as deteriorated and fragmented *M. leprae*. The dense materials are also found in the cytoplasm of vascular endothelial cells. In lepromatous cells both Schwann and endothelial cells frequently harbor *M. leprae* (25). The nerves are progressively destroyed and replaced by fibrous tissue, in both paucibacillary and multibacillary cases (27).

The peripheral nerve damage in leprosy often results in sensory and motor dysfunctions that lead to permanent deformities and/or disabilities (28). Innate immune and inflammatory genes were modulated by *M. leprae* during early infection (29). Therefore, the understanding of the innate immune pathways in the local of infection is crucial for the development of new strategies to control leprosy and its reactional episodes (Table 1).

**Table 1:** Innate immunity-modulating strategies and possible therapeutic targets.

Targets	Therapeutic strategies	Reference
TLR2	Vaccine	(30, 31)
	Vaccine adjuvant	(32)

TLR4	Vaccine	(33, 34)
	Vaccine adjuvant	(35, 36)
	Adjuvant immunotherapy	(37, 38)

TLR9	Vaccine adjuvant	(39, 40)

NOD1	Immunostimulant therapy	(41, 42)

NOD2	Immunotherapy	(43)
	Vaccine adjuvant	(44)

Bcl-2	Induction of apoptosis	(45, 46)

TNF	Inhibition of TNF cytokine effects	(47, 48)

Autophagy	Vaccine	(49)
	Pathogen replication control	(50)
	Restriction of mycobacteria growth	(51–57)

## Innate Immune Cells in Leprosy

The use of monoclonal antibodies to label specific membrane antigens is one of the most used tools to identify the presence and the frequency of different cell populations in tissue. Several studies demonstrated an enormous diversity in cell phenotypes present in different tissues. The proportions of each cell type amongst the total population of non-lymphoid mononuclear cells are different in the various leprosy infiltrates (58). In addition, the characterization of different cell phenotypes in dermis and epidermis has been shown by many studies (16, 59–61).

Despite the existence of predominant macrophage phenotypes well described in literature, between the polar forms of leprosy, it is widely recognized that some terminologies are simplistic and cells like macrophages may present a broad spectrum of differentiation states, continuously regulated by a myriad of signals from the microenvironment (62, 63). In conjunction of Th1–Th2 dichotomy, macrophages have been classified in M1 and M2. Stimulation with proinflammatory cytokines as interferon (IFN)-γ activate M1 macrophages, characterized by enhanced antimicrobial, inflammatory, and antigen-presenting properties, whereas cytokines like interleukin (IL)-4 and IL-13 activate M2 macrophages, which portray anti-inflammatory actions, being associated with tissue repair and fibrosis (62, 63).

Our previous study has demonstrated that in skin cells from lepromatous patients that developed reversal reaction there is a coexistence of M1 and M2 populations in the midst of the inflammatory environment, together with a wide diversity of DC subsets (15, 64). The hallmark of the reversal reaction has been broadly accepted as the appearance of immature and loose epithelioid granulomas, which differ from the typical mature epithelioid granuloma seen in the TT forms. The epithelioid cell is described as a non-phagocyte of unknown ontogeny with high secretory capacity that could be a differentiation state of skin macrophage populations (65, 66). Facchetti et al. (67) described a cell type they called plasmocytoid monocytes (PM) and suggested, based on ultrastructure and immunohistochemical data, that they are the precursors of the epithelioid cells (68). These cells’ phenotypic profile includes DCs and macrophage markers, being identified as a CD3^−^, CD11c^−^, CD14^−^, CD20^−^, CD36^+^, CD56^−^, CD68^+^, CD123^+^, BDCA2^+^ population (69). Since PMs produce high levels of type I IFN and express CD123, they are also thought to be a previous immature state of the plasmocytoid DC (pDC) (67).

Although efforts to identify cell markers and inflammatory mediators *in situ* the immunopathogenesis of leprosy is not fully understand. The high heterogeneity and the existence of mixed cell phenotypes in different timepoints of infection that are influenced by the mediators produced in tissue microenvironment together with the inexistence of antibodies highly specific to clearly differentiate human cells contribute to the difficulty of establish a precise role of each cell type in leprosy immunopathogenesis.

### Macrophages

Macrophages have been identified as key players in the pathogenesis of leprosy. It has been demonstrated that during an inflammatory response, bone marrow derived monocytes enter the tissue in large numbers and take part in the defense against the pathogens. In a very elegant study, Kibbie et al. (70) demonstrated that unstimulated endothelial cells trigger monocytes to become M2 macrophages and that IFN-γ activates endothelial cells to induce monocyte to differentiate into M1 macrophages by a mechanism regulated by Jagged 1 (JAG1), a protein localized in the vascular endothelium. It is known that tissue macrophage populations have a mixed embryonic and postnatal bone marrow origin, but the exact mechanisms of differentiation and activation is not understood. There are a lot of evidences that a significant percentage of tissue macrophages are independent from blood monocytes and different phenotypes or functions are the result of different macrophages origin (71). Therefore, it is not possible to differentiate resident-tissue macrophages and recruited monocyte-macrophages once they coexist in a common environment (63).

The heterogeneity of tissue resident macrophages during homeostasis and inflammation shows that macrophages cannot be correctly classified as M1 or M2 when in a specific tissue. Although too simplistic, this nomenclature has been used in order to establish the pivotal role of macrophages in the establishment of the different forms of the disease. Each clinical presentation in leprosy is associated with a different macrophagic population in host tissue. Macrophages can present a proinflammatory M1 phenotype in which vitamin D-dependent antimicrobial pathway predominates, as observed in the paucibacillary lesions and in the onset of reversal reaction (72, 73); through to anti-inflammatory M2 phenotype in which there is an upregulation of phagocytic pathways as found in lepromatous skin tissues (72–75).

Immunohistochemistry analysis demonstrated a high expression of Galectin-3 on macrophages found in skin lesions of lepromatous patients; in contrast, it is almost undetectable in tuberculoid lesions. The increase of Galectin-3 in lepromatous cells contributes for reduced T cell activation in these patients (76).

de Sousa et al. (74) have demonstrated that the understanding of the role of cytokines, arginase 1, and costimulatory molecules in macrophages may contribute for the comprehension of innate immunity function in the establishment of the polar forms of leprosy. In addition, Teles et al. demonstrated that in macrophages present in lepromatous skin cells there is an upregulation of IL-27 (77), a paradoxal cytokine that may activate IFN-β and IL-10 that contribute for the blockade of antimicrobial pathways (78).

**Figure 1:**
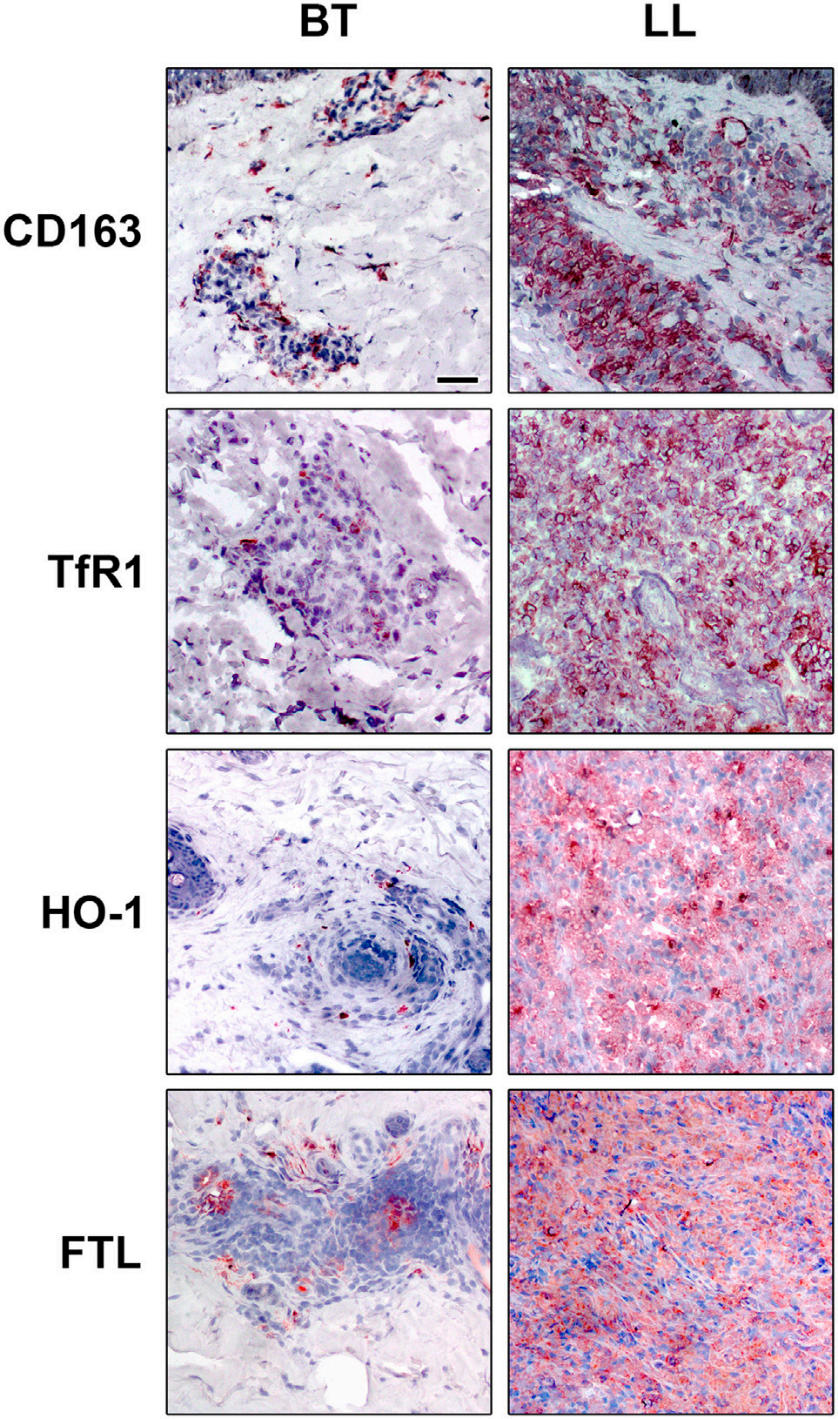
Iron-related proteins are differentially regulated in leprosy clinical forms. Lepromatous skin lesions [lepromatous lepromatous (LL)] present a higher expression of the scavenger receptor of hemoglobin–haptoglobin complex, CD163 (upper panels), transferrin receptor 1 (TfR1, mid upper panels), the enzyme that catalyzes heme, heme-oxygenase 1 (HO-1, mid lower panels), and of the iron storage protein ferritin [ferritin light chain (FTL) lower panels]. The protein expression of CD163, TfR1, HO-1, and FTL was evaluated by immunohistochemistry. Images are representative of five independent samples from each group. Scale bars: 50 mm.

Although the predominance of specific cell markers of M1 or M2 in the different clinical forms of leprosy, there is a continuum of phenotypes between these ranges with some cells sharing phenotypes of both M1 and M2 macrophages. Lepromatous macrophages, while predominantly expressing M2 markers like CD163, indoleamine 2,3 dioxygenase (IDO), arginase, and SRA-I (16, 61, 79–81), have some M1 characteristics like increased iron storage and a diminished expression of the iron exporter Ferroportin (Fpn-1) as well, which indicates that augmented iron deposits may favor *M. leprae* survival inside the foamy macrophages (79) (Figure 1).

Besides iron, *M. leprae* incorporates cholesterol and converts it to cholestenone; however, it does not use cholesterol as a nutritional source (82), although cholesterol colocalizes to *M. leprae*-containing phagosomes, and the blockade of cholesterol decreases the bacterial survival (83). Previous studies have demonstrated that *M. leprae* induces lipid body biogenesis and cholesterol accumulation in host cells (84). In lepromatous lesions, host-derived oxidized phospholipids were detected in macrophages, and one specific oxidized phospholipid, 1-palmitoyl-2-(5, 6-epoxyisoprostane E2)-sn-glycero-3-phosphorylcholine accumulate in macrophages infected with live mycobacteria (85). Normal HDL, a scavenger of oxidized phospholipids, may revert the inhibition of innate immune responses caused by mycobacterial infection. However, this effect was not observed when they have used HDL from lepromatous patients (85).

### Dendritic Cells

In skin, DCs are located in the epidermis, as Langerhans cells and in the dermis, as dermal DCs (59). Langerhans cells in leprosy skin lesions express CD1a and langerin. These cells efficiently present antigens to T cells as part of the host response to *M. leprae* (86).

Previous reports have demonstrated that Langerhans cells are dendritic cells; however, recent findings that evaluate the transcriptional profile have suggested that Langerhans cells may be considered resident macrophages (87, 88). Since several published studies classified these cells as dermal dendritic cells, we maintain this definition in this review.

CD1a^+^ cells are associated with the outcome of reactional episodes in leprosy (89). CD1a is expressed in CD123^+^ cells located in the dermis from both lepromatous and reversal reactional patients (15). Quantitative analysis showed a clear predominance of dendritic cells in tuberculoid leprosy (80, 89–91), whereas lesions from patients with the lepromatous pole of the disease show weak induction of CD1 proteins (89, 90). This weak expression of CD1 in lepromatous lesions is not result to a primary defect of the CD1 system itself because CD1a, CD1b, and CD1c could be induced to similar levels in both tuberculoid and lepromatous monocytes. Therefore, local factors at the site of infection may be responsible for the blockade of CD1 expression in lepromatous cells (90).

In lesions from tuberculoid leprosy patients, dendritic cells were linked with matrix metalloproteinase (MMP)-12 and contribute to granuloma formation (75). Previous studies have demonstrated that IDO-1 expression in myeloid dendritic cells and macrophages are part of the immune response associated with granuloma formation and may be associated with the granulomatous reactions in the skin (92). Our previous study has demonstrated that in lepromatous lesions IDO^+^ cells with a dendritic-like morphology are detected on the dermis and in some endothelial cells (16). The characterization of IDO^+^ cell phenotype demonstrates that almost all cells constituting the lepromatous dermal infiltrate are positive for HLA-DR, CD11c, CD86, and CD68. In tuberculoid lesion a few cells are positive for IDO and CD11c^+^ and CD86^+^ cells are detected in the center of the granuloma probably corresponding to epithelioid macrophages (16). In lepromatous patients that develop reversal reaction an increase in IDO gene expression is observed (15). The morphological changes in the reversal reactions skin lesions are accompanied by phenotypic heterogeneity of myelomonocytic populations. The epithelioid cells exhibit both DC and macrophage markers, hinting at the complexity of this cell type. These cells found in the reversal reaction granuloma are CD68^+^, CD83^+^, CD206^+^, CD209^+^, CD1b^+^, CD11c^+^, and CD123^+^, but did not express CD163. Double-immunofluorescence data also show that these cells express BDCA2 and BDCA4, suggesting that define pDC populations.

*Mycobacterium leprae* components trigger CD209 on DCs to induce IL-10 production in lepromatous cells (93). In addition, CD209 may function as a receptor of entry for *M. leprae* in host cells (94). The dendritic cells phagocytose *M. leprae* and express antigens derived from the bacteria, such as phenolic glycolipid 1 (PGL-1). Hashimoto et al. (95) demonstrated that *M. leprae* infection decreases the capacity of DCs in inducing T-cell responses by a mechanism that involves PGL-1, since the blockade of PGL-1 in the surface of DCs enhanced CD4(+)- and CD8(+)-T-cell responses. Other studies have also demonstrated that PGL-1 impairs dendritic cells maturation and activation, thereby facilitating *M. leprae* survival (96, 97).

### Keratinocytes

The response of the epidermis to *M. leprae* infection can be shown by the different aspects seen along the spectrum as well as during reactional states. The epidermis plays an important role in the local inflammatory response detected in leprosy. Keratinocytes expressing ICAM-1 are found in lesions from leprosy patients that present strong cellular immune response against *M. leprae* (tuberculoid, reversal reaction), but not in lepromatous lesions (98). PCR analysis demonstrated the expression of inflammatory cytokines TNF, IL-6, and IL-12 besides high expression of ICAM-1 in the epidermis of reactional leprosy lesions (99).

Keratinocytes are more susceptible to *M. leprae* infection than dendritic cells that spontaneously present higher concentrations of the antimicrobial peptide cathelicidin (100). Previous study demonstrated an up-regulation of human beta-defensins 2 and 3 (hBD2 and hBD3) in keratinocytes stimulated with *M. leprae*, which is reverted by corticosteroids. In addition, they have demonstrated that corticosteroid treatment of patients with reversal reactions causes a suppression of hBD2 and hBD3 in skin biopsies, as measured by qPCR (101).

The role of keratinocytes during the reactional episodes needs to be better evaluated since besides their possible role in reversal reaction, these cells may be involved in the pathogenesis of ENL. It was demonstrated that Thalidomide therapy downregulates the expression of ICAM-1 and HLA-DR antigens in keratinocytes (102).

HLA-DR^+^ keratinocytes could present *M. leprae* antigens to well-defined CD4^+^ cells (103). However, increased keratinocyte expression did not represent a control of bacillary load since recombinant granulocyte–macrophage colony-stimulating factor (GM-CSF) administered intradermically or by subcutaneous route leads to enhanced keratinocyte growth but the bacillary numbers remain unchanged (104). In tuberculoid skin lesion cells keratinocytes are the major producer of CXCL-10 but not in lepromatous cells (105), probably because it is necessary IFN-γ produced by T cells to induce this chemokine.

### Schwann Cells

*Mycobacterium leprae* may cause peripheral neuropathy. *M. leprae* is able to overcome a succession of physical barriers—epineurium, perineurium and endoneurium—until it reaches the Schwann cell, taking advantage of the difficulty of immune cells to access these impervious barriers (106–108). *M. leprae* may infect both myelinating and non-myelinating Schwann cells in patients with leprosy, but *M. leprae* preferentially infects the non-myelinating Schwann cells (109). There is not a consensus if the neural damage is a result of *M. leprae* entry inside cells or it occurs because of the inflammatory infiltrate.

Masaki et al. (110) demonstrated that *M. leprae* may generate dedifferentiated Schwann cells by causing initial demyelination to establish infection, colonize the cells, and subsequently reprogram them to a progenitor/stem cell-like cells (pSLCs) stage to spread the infection. In addition to downregulating Schwann cell lineage transcripts and reactivating developmental genes, *M. leprae* induce a large numbers of immune-related genes comprising mostly innate immunity from the very early stage of Schwann cell infection and peaking in their expression when Schwann cells have changed their cell identity to pSLCs (29). A previous study demonstrated that *M. leprae* could modulate Schwann cell glucose metabolism to increase the generation of the reduction capacity and free-radical control (111), but the impact of these regulation in nerve damage needs to be more clarified.

Schwann cells in skin lesions from leprosy patients express TLR2 (112, 113). In nerve biopsies from patients with neuritis, it was identified TNF, TNF receptors and TNF-converting enzyme in Schwann cells (26). It was speculated that *M. leprae* ligands induce Schwann cell death by a pathway that involves both TLR2 and TNF. It is possible that the pro inflammatory cytokines may contribute for Schwann cell apoptosis after cell interaction with *M. leprae*, which is associated with the pathogenesis of nerve damage (112, 113).

Analysis of nerves of pure neuritic patients demonstrated that MMP-2, MMP-9, and TNF mRNA production is highly induced in the AFB(−) lesions in relation to AFB(+) neuritic leprosy and non-leprosy control group (114), whereas CCL2 and CXCL10 chemokines are not determinant for the establishment of AFB(+) or AFB(−) in advanced stages of leprosy nerve lesions. CCL2 expression is associated with macrophage recruitment and fibrosis (115).

Recent findings have demonstrated that nerve damage begins in the early stages of the disease and may be more strongly related to response of innate immunity. In this context, the complement system has been placed with relevant role. This system is part of the innate immunity against bacterial pathogens through the formation of Membrane Attack Complex (MAC), but can lead to an inflammatory process followed by tissue injury if activated uncontrollably. Histopathological studies demonstrated MAC deposition on cutaneous sensory nerves (116) and on damaged nerves of lepromatous patients. However, the same was not found for tuberculoid patients (117).

Advancing in studies related to the complement system as a trigger for nerve damage, a pathogen-associated molecular pattern (PAMP), the glycolipid lipoarabinomannan (LAM), a component of the mycobacteria cell wall, has been investigated as the starting mechanism for activation of this pathway. It has been shown *in vitro* that this PAMP activates the Schwann cell by the formation of opsonin C3 and MAC (118). In nerve biopsies of leprosy patients, in turn, the LAM and MAC antigen deposition was found. MAC and LAM colocalizes on axons suggesting a relation between LAM in complement activation and nerve damage (117). In a mouse nerve lesion model, the interaction of LAM with the nerve was observed, activating the pathway of the complement system (117).

Recent evidences suggest that axon demyelination occurs in function of the interaction of PGL-1 with myelinating glia and their infection. According to Madigan et al. (119) demyelination and axonal damage are initiated by infected macrophages that patrol axons. PGL-1 induces nitric oxide synthase in infected macrophages that results in damaged axons by injuring their mitochondria and inducing demyelination (119).

### Neutrophils

Little attention has been given to the function of the neutrophils in leprosy. It was previously demonstrated that both circulating neutrophils and monocytes are loaded with intracellular *M. leprae* without obvious inflammatory phenomena (120, 121). We reported that neutrophils isolated from lepromatous leprosy patients with or without ENL release TNF and IL-8 after stimulation with *M. leprae* (122). Moreover, the apoptotic rate of ENL neutrophils is higher as compared to lepromatous patients and healthy volunteers (122).

Microarrays analyses comparing skin lesions of lepromatous patients and patients with ENL revealed the up-regulation of cell movement genes, including E-selectin and its ligands, key molecules that mediate neutrophil recruitment to inflammatory sites (123). According to these results “granulocyte adhesion and diapedesis” were identified by Dupnik et al. (124) as one of the top canonical pathways characterizing ENL. Moreover, neutrophil and endothelial cell gene networks were identified in ENL samples as part of the vasculitis that results in tissue injury (75).

Recently, we reported that during ENL, but not in RR, circulating neutrophils express CD64 on cell surface, while nonreactional leprosy or healthy volunteers have lower levels of CD64 expression. CD64 expression on circulating neutrophils and in ENL lesion is down modulated after thalidomide treatment. Moreover, the severity of ENL is associated with high levels of CD64 expression, also pointed as an early biomarker for ENL (20). Increased CD64 expression *in vivo* has been associated with an increase in neutrophil function and adhesion to the endothelium (125–128).

Elevated levels of TNF and other proinflammatory cytokines have been associated with episodes of ENL, while suppression of TNF leads to clinical improvement (102, 129). We reported evidence that pentraxin-3 (PTX3), originally described as a protein induced by primary inflammatory signals, such as TNF and IL-1β, is released systemically and at the site of ENL lesions (130). We also demonstrated that there is a positive correlation between PTX3 serum levels and CD64 surface expression on circulating neutrophils. Moreover, we showed that the majority of neutrophils (MPO^+^ cells) presented throughout the ENL lesion express PTX3 (130). Additionally, thalidomide treatment of ENL downregulated PTX3 levels. Interestingly, PTX3 serum levels were higher in lepromatous patients without reaction that developed ENL, persisting after the onset. In contrast, lepromatous patients that developed reversal reaction had lower levels of PTX3 prior and during the inflammatory episode. Those data indicate that high levels of PTX3 may be associated with ENL occurrence and point to PTX3 as a potential ENL biomarker able to differentiate from a reversal reaction episode. Belone et al. (131) previously reported the PTX3 mRNA is exclusively expressed in ENL lesions.

## Manipulation of Innate Immunity by *M. leprae*

To survive within the host cell, mycobacteria must escape intracellular mycobactericidal mechanisms.

The activation of innate immunity may occur after the interaction of PAMPs, which are conserved microbial structures, with their respective pattern recognition receptors (PRRs), present in host cells. PRRs are also able to recognize endogenous molecules from damaged cells, known as damage-associated molecular patterns (DAMPs), resulting in several chronic inflammatory and autoimmune diseases. After the interaction of PAMPs and/or DAMPs with PRRs, the release of intracellular signals leads to the induction of important genes transcription for cellular activation or induction of phagocytosis. Different PRRs are expressed in the same cell, which makes it able to recognize several classes of microorganisms and different endogenous molecules. The PRRs described so far are C-type lectin receptors, Nod-like receptors (NLRs), RIG-1-like receptors, and toll-like receptors (TLRs) (132–134).

Complement activation, apoptosis, and autophagy are other innate mechanisms modulated by the mycobacteria. The understanding of the mechanisms and pathways used by mycobacteria to manipulate the innate immunity may contribute for the development of new strategies of diagnostic and control of the disease.

### Toll-Like Receptors

Several studies indicate that the recognition of mycobacteria by TLRs represents an essential step in generating an immune response capable of protecting the infection.

Different molecules that constitute *M. leprae* have been characterized as ligands and potent stimulators of TLRs, mainly involving TLR2. Killed *M. leprae* is able to mediate TLR2/1 heterodimers and TLR2 homodimers cell activation, indicating the presence of triacylated lipoproteins in the bacterium (135). In fact, a genome-wide scan of *M. leprae* identified 31 lipoproteins with potential to act as ligands of TLR2/1 heterodimer (135). As *M. leprae* cannot be grown *in vitro*, the purification of proteins from the few bacteria in armadillos becomes very difficult. Therefore, Krutzik et al. (135) used synthetic lipopeptides to show that the 19 and 33-kDa lipoproteins from *M. leprae* are capable to activate *in vitro* both monocytes and dendritic cells. In addition, lesions from leprosy patients with localized tuberculoid form displayed more strongly expression of TLR2 and TLR1 as compared with the lepromatous form of the disease. These data suggest the involvement of TLRs in the host defense against the mycobacteria.

Nerve damage is an important clinical hallmark of leprosy disease responsible for the patient morbidity. In this sense, the activation and expression of TLR2 have also been investigated in human Schwann cells (112). The lipopeptide that mimics the *M. leprae* 19-kDa lipoprotein, and can act as TLR2 agonist, induced an increase in the number of apoptotic cells during activation of Schwann cells (112). It was possible to identify the expression of TLR2 in Schwann cells present in lesions from tuberculoid patients, in addition to cells that had undergone apoptosis *in vivo* (112), providing a link between innate immune response and nerve injury in leprosy.

The presence of foamy cells highly infected is characteristic in lepromatous, but not in tuberculoid lesions. The foamy phenotype results from the capacity of *M. leprae* to induce and recruit host-derived lipids to bacterial-containing cells, forming lipid droplets (12). Interestingly, TLR6 is essential for lipid droplets biogenesis in *M. leprae*-infected Schwann cells, but not TLR2 (136). On the other hand, the formation of lipids droplets in *M. leprae*-bearing macrophages appeared to be only partially dependent on both TLR2 and TLR6 (12). These data suggest the involvement of alternative TLRs or additional receptors associated with the innate immune response for *M. leprae* recognition in leprosy.

Polycarpou et al. (137) demonstrated that *M. leprae* activates TLR4, by containing uncharacterized ligands, since the classic ligand agonist of TLR4 is LPS (138). TLR4 neutralizing antibody pretreatment decreased the production of TNF, IL-6, and CXCL-10 in human macrophages stimulated with *M. leprae* (137). Furthermore, *M. leprae* upregulates TLR4 protein expression on macrophages from healthy subjects, but not in macrophages from BCG-vaccinated donors (137). Macrophages from non-vaccinated healthy donors treated with BCG present reduced TLR4 expression suggesting a role of TLR in the protective effect of BCG. Associated with this, the treatment of reversal reaction with corticosteroids decrease gene and protein expression of both TLR2 and TLR4 in skin lesion cells (139), indicating the involvement of receptors also in triggering the inflammatory process. A study linking the innate immunity pathways with the development of ENL suggested that recognition of DNA by TLR9 constitutes a major inflammatory pathway activated during ENL (140). The proinflammatory cytokines storm observed during ENL seems to be related to the massive release of mycobacterial TLR9 ligands during multidrug therapy (140). Moreover, the inflammatory response could be amplified by the binding of endogenous DNA to TLR9 (140), since expressive tissue destruction also occurs during ENL (141). Dias et al. (140) demonstrated a higher TLR9 expression in cells from ENL patients when compared with nonreactional lepromatous controls. In addition, significantly increased circulating levels of human and mycobacterial DNA–histone complexes were detected in ENL patients when compared with nonreactional controls (140). Furthermore, TLR9 agonists were able to induce the secretion of higher levels of TNF, IL-6, and IL-1β in ENL when compared with nonreactional patients and healthy individuals (140). The same effect was observed in the cells stimulated with lysed *M. leprae* (140). The use of a synthetic antagonist of nucleic acid-sensing TLRs suggested that this may be an alternative for the development of more effective drugs to treat ENL.

The genetic association demonstrated several single-nucleotide polymorphisms (SNPs) in TLR genes that may be associated with susceptibility or resistance to leprosy and leprosy reactions. However, most studies in this area focused mainly on the mutation of TLRs 1 and 2 and their correlation with the disease. The SNP within TLR1 (I602S) is associated with reduced responses to mycobacterial agonists (142). The TLR1 602S variant, but not the TLR1 602I variant, in heterologous systems showed the expected absence of the receptor on the plasma membrane (142). The 602S allele is associated with a reduced incidence of leprosy (142).

Previous studies showed that TLR1 variants N248S is a susceptibility factor for leprosy (143, 144). Additionally, PBMCs from individuals carrying 248S produce a lower TNF/IL-10 proportion levels after stimulation *in vitro* with *M. leprae*, but not with controls as LPS (TLR4 agonist) or PAM3CSK4 (TLR2 agonist) (144). Analysis of samples from patients that developed reactional episodes demonstrated that a TLR1 N248S-linked feature is associated with the development of disabilities and the progression from infection to disease (143).

Another transmembrane domain polymorphism in TLR1 (T1805G) was associated with susceptibility to leprosy, regulating the innate immune response (145). The group analyzed 933 Nepalese leprosy patients, 238 of whom with reversal reaction, and investigated the association of TLR1 variation with different clinical forms of leprosy or reversal reaction, demonstrating that the1805G allele is associated with protection from reversal reaction (145).

A TLR2 mutation in the lepromatous, but not in tuberculoid patients, was also identified (146). TLR2 from PBMCs from lepromatous patients presented a C to T substitution at nucleotide 2029 from the start codon. This modification was not identified in tuberculoid individuals (146). In fact, periphery monocytes from leprosy patients with modification in TLR2 (Arg677Trp) were significantly less responsive to cell lysate of *M. leprae* than subjects carrying wild-type TLR2 (147). Additionally, the secretion of IL-12 was lower in patients with TLR2 mutation (147). A study performed in Ethiopian patients investigated different polymorphisms in TLR2 (597C→T, 1350T→C, and a microsatellite marker) (148). The mutation-associated risk of developing leprosy was assessed. The microsatellite and the 597C→T polymorphisms were both associated with susceptibility to reversal reaction as predicted by reversal reaction.

The roles of TLR1 and 2 in leprosy and leprosy reactions were described and it may contribute for perspectives in leprosy management.

### NLRs

The nucleotide-oligomerization domain (NOD) proteins are intracellular and cytoplasmic receptors. Previous data have demonstrated that the blockade of phagocytosis inhibits IL-1β and TNF production in response to *M. leprae*, suggesting that intracellular signaling is also required for macrophage activation after *M. leprae* infection. In addition, NF-κB activation and expression of TNF and IL-1β were observed in NOD1- and NOD2-transfected cells stimulated with *M. leprae* (149).

NLRPs are intracellular receptors that recognize PAMPs and induce the secretion of both caspase-1 and IL-1β in the context of inflammasome. SNPs in NLRP1 and NLRP3 genes were analyzed in Brazilian leprosy patients. The NLRP1 combined haplotype rs2137722/G-rs12150220/T-rs2670660/G was significantly more frequent in patients than in controls as well as in paucibacillary than in multibacillary patients (150). The NLRP1 combined haplotype rs2137722/G-rs12150220/A-rs2670660/G was associated with paucibacillary leprosy suggesting that NLRP1 might be involved in the susceptibility to leprosy (150).

Nod-like receptors may recruit and activate inflammatory caspases into inflammasomes or may trigger inflammation *via* different pathways including the NF-κB mitogen-activated protein kinase and regulatory factor pathways (151).

Polymorphisms in NOD2 are associated with leprosy susceptibility. Activation of monocytes *via* NOD2 induces preferentially the differentiation into dendritic cells, which was mediated by IL-32. Notably, IL-32 is able to induce monocytes from healthy donors or from tuberculoid patients to rapidly differentiate into DCs, which is more efficient than GM-CSF-derived DCs in presenting antigen to major histocompatibility complex class I-restricted CD8(+) T cells (152). In contrast, monocytes from patients with the lepromatous form of leprosy did not produce IL-32 in response to NOD2L and did not induce DC differentiation by a mechanism that is mediated by IL-10 (152). In tuberculoid patients there was a higher expression of NOD2 and IL-32 as well as the frequency of CD1b (+) DCs at the site of leprosy infection when compared with lepromatous patients (152, 153).

### Complement Cascade

Lipoarabinomannan is a molecule from *M. leprae* that is associated with nerve damage. Curiously, previous studies demonstrated that LAM activates complement and previous study demonstrated the important role of complement in nerve damage in leprosy (117). Analysis of skin biopsies demonstrates that the percentage of CD3d, MAC, and LAM deposition is significantly higher in lepromatous when compared to tuberculoid patients (154). MAC deposition colocalizes with LAM and is found on axons in skin lesions of lepromatous patients. In tuberculoid lesions, the presence of T cells positive for CD3d was observed in surrounding granulomas without MAC deposition (154). Analysis of skin lesions from reactional patients demonstrated an increase in MAC immunoreactivity when compared to non-reactional leprosy patients (154). Immunofluorescence analysis showed an increase of C1q deposition in both reversal reaction and ENL lesions when compared to non reactional matched patients (124).

Lahiri et al. demonstrated that when disrupted, *M. leprae* could activate complement (155) and polymorphisms in genes of complement cascade suggest an association of complement genes with leprosy susceptibility (156).

### Apoptosis

Analysis of skin lesion cells demonstrated that apoptosis is more frequent in tuberculoid and reversal reaction than in lepromatous cells (157–159). Lepromatous cells present increased expression of the antiapoptotic protein Bcl-2, suggesting that the decrease in cell death could contribute for sustains the infection (158).

The hypothesis of the involvement of apoptosis in the control of bacillary load was reinforced by *in vitro* studies that demonstrated that clofazimine, a compound used for the treatment of leprosy since the 1960s has the capacity to induce apoptosis in macrophages, suggesting that the antibacterial and anti-inflammatory properties of this drug are mediated by apoptosis (160). Analysis of apoptosis in skin cells from treated patients revealed that in both tuberculoid and lepromatous lesions, there is an increase in the frequency of apoptotic cells at 3 and 6 months after the start of the treatment (161). Analysis of lesions in either reversal reaction or ENL demonstrated a significant increase in apoptosis only in ENL lesions and those that were at 6 months of treatment (161).

Although several studies suggesting the antibacterial role of apoptosis in infected cells, there are evidences that in tuberculoid patients apoptosis is a mechanism that contributes to maintain the infection, instead of the pro inflammatory infiltrate and the presence of pro inflammatory cytokines. In tuberculoid lesions predominate a M1 phenotype, although few M2 cells were present in the skin lesions of these patients (16, 61). We have previously demonstrated that *in vitro* GM-CSF-differentiated monocytes (M1) stimulated with both *M. leprae* and apoptotic cells change their phenotype and express M2 cells-specific markers, such as CD163 and SRA-I. Moreover, the phagocytosis of apoptotic cells by *M. leprae*-infected macrophages increases the secretion of anti-inflammatory mediators as IL-10, TGF-β, and arginase, corroborating the hypothesis that in paucibacillary patients, although the presence of an effective cellular immune response, efferocytosis contributes for maintain few susceptible macrophages in skin lesions which contributes for sustain the infection (81).

The induction of apoptosis in Schwann cells stimulated with *M. leprae* was previously demonstrated (112, 113) and some studies associated apoptosis in Schwann cells as an important event for nerve damage. *M. leprae* induces demyelization in Schwann cells by a pathway that involves the activation of the MAPK (ERK 1/2). A previous study has demonstrated that the ganglioside 9-O-acetyl GD3 is associated with *M. leprae* entry in Schwann cells and that the blockade of this ganglioside may result in a reduced activation of the MAPK (ERK 1/2) pathway (162).

### Autophagy

The canonical macroautophagy (hereafter termed autophagy) pathway is an evolutionarily conserved mechanism through which organelles and proteins are degraded and recycled by the lysosomal system to promote cellular and organismal homeostasis. The major hallmark of autophagy is the formation of double-membrane vesicles called autophagosomes, which engulf and driving intracellular targets for degradation. Autophagy impairment is widely implicated in the pathology of several diseases, including microbe infection, cancer, and metabolic, cardiovascular, and neurodegenerative disorders (163).

During infectious processes, autophagy helps the immune system by degrading intracellular microbes through a selective form of autophagy called xenophagy. The significance of autophagy in numerous infectious processes was established, including those caused by bacterial, parasitic, and viral pathogens, as well as the microbial strategies used to avoid or subvert autophagy and promote their own survival (164, 165). In contrast, the role of autophagy in leprosy pathogenesis remained unknown until recently. The first evidence that *M. leprae* can be targets for autophagy was revealed by transmission electron microscopy studies. It was observed that during the initial growth phase of *M. leprae* in macrophages, the mycobacteria are present in single membrane vacuoles with few nearby lysosomes, and the bacilli are intact. At the peak of the growth phase, the number of lysosomes increases and *M. leprae* is located in a large number of double membrane vacuoles.

During the stationary phase, macrophages have a vacuolar appearance and contain a significant number of lysosomes, *M. leprae* organisms are inside double membrane vacuoles, and most of these bacteria are degenerate (166). Chandi and Job (167) described the presence of double membrane phagosomes in macrophages after 40 min of *M. leprae* exposure, and after that, the lysosomes fuse with these *M. leprae*-containing vacuoles. These data provide evidences that *M. leprae* may have been the first bacterial pathogen to interact with the autophagic pathway and reinforces the role of autophagy in leprosy.

A genomewide association study of leprosy revealed that a polymorphism in the upstream autophagy activator gene NOD2 is a susceptibility factor to develop *M. leprae* multibacillary infection (168, 169). Interestingly, the polymorphisms in other autophagy-associated genes such as PARK2, VDR, and TLR2, are also correlated with the multibacillary leprosy susceptibility (51, 146, 170–174). In other hand, these triggers of autophagy are preferentially expressed in the skin lesions of the auto limited tuberculoid clinical form (72, 135, 152). Subsequently, it has been suggested that the polymorphism in the autophagy gene IRGM, which is linked to susceptibility to Crohn’s disease and tuberculosis (175–178), is associated with an increased risk of developing leprosy because it affects the production of inflammatory cytokines such as IFN-γ (179). In addition, increased IRGM expression was observed in monocytes and macrophages infected with *M. leprae*, as well as, monocytes from the self-limited tuberculoid form presented a higher expression of IRGM, as compared to cells of clinically progressive lepromatous patients (180). IRGM, an effector of IFN-γ-mediated autophagy, controls the autophagic pathway through their interaction with ULK1 and BECN1, governing the assembly of the initiation complex, and then together with ATG16L1 and NOD2, forms a molecular complex that promotes antimycobacterial defense (181, 182).

More recently, our group described an association between *M. leprae* death and targeting of mycobacteria to the autophagic pathway in human macrophages. It has been shown that the genetic silencing of the OASL antiviral protein, which is produced through the detection of *M. leprae* DNA mediated by STING sensor, increases the levels of autophagy and decreases the viability of the mycobacteria, being reversed by the autophagy blockade (183). Ma and cols (184) suggested that although autophagy could promote the elimination of intracellular pathogens, the induction of the autophagic pathway by *M. leprae* would be a mycobacteria pro-persistence factor. It has been reported that although activation of autophagy occurs in response to *M. leprae* infection in macrophages, it also promotes an IL-10-producing T cell-mediated anti-inflammatory response, which in a negative feedback cycle inhibits autophagy and allows *M. leprae* survival in macrophages (184). However, this work was based only on the alone use of CYTO-ID/CAT, an acidotropic dye from the monodansylcadaverine group recently developed to monitor autophagy in living cells (185), which is not recommended by autophagy experts (186).

**Figure 2:**
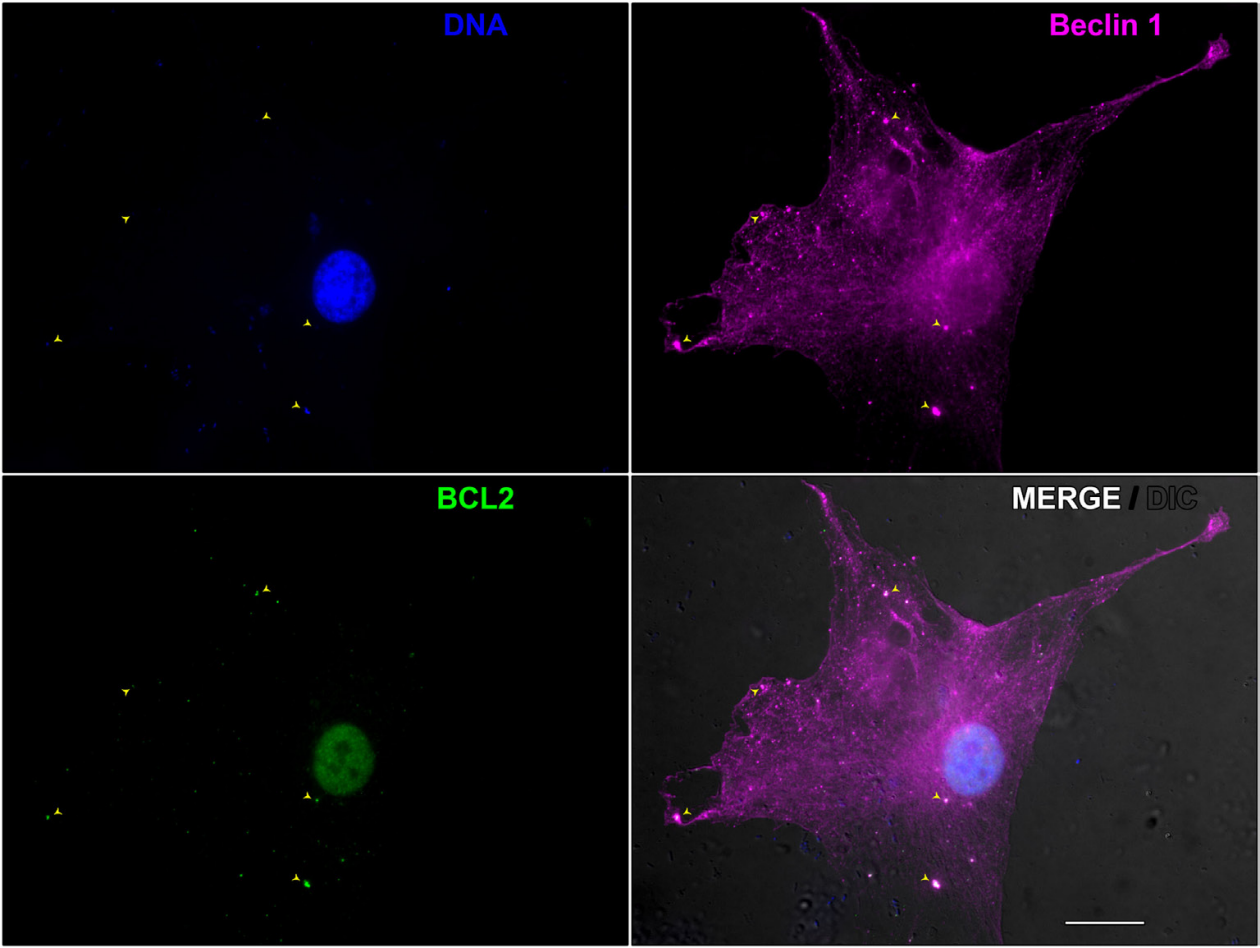
Beclin 1-mediated autophagy during *Mycobacterium leprae* infection in skin lesion-derived lepromatous macrophage. Macrophages were isolated from the skin lesion of a lepromatous leprosy patient and cultured for 18 h in full nutrient medium. Cells were fixed and immunofluorescence for Beclin 1 (magenta) and BCL2 (green) was performed. Cellular and bacterial DNA were stained with DAPI (blue). Cell and mycobacteria morphology are shown by Nomarski differential interference contrast (gray). This image shows a lepromatous tissue macrophage interacting with *M. leprae*. BCL2 colocalizes with Beclin 1-entrapped mycobacteria (arrowheads) allowing *M. leprae* survival through autophagy inhibition. The modulation of autophagy has the potential to be useful in the leprosy treatment, as well as to prevent leprosy reactional episodes. Scale bar: 20 µm.

Finally, we demonstrated the key role of autophagy in leprosy polarization (187). We showed that autophagy is differentially regulated between leprosy polar forms, uncovering an essential role for Beclin 1 protein in this process, which was upregulated in tuberculoid patients. In contrast, a higher expression of BCL2 protein was determined in lepromatous patients. While Beclin 1 is a key initiator of the functional formation of autophagosomes in mammals and may be induced by IFN-γ to activate autophagy, the BCL2 antiapoptotic protein inhibits autophagy by binding and sequestering Beclin 1 from the class III PIK3 complex (188). In tuberculoid skin lesion cells IFN-γ-induced autophagy contributes for *M. leprae* control, whereas in lepromatous cells the BCL2-mediated block of the Beclin 1 autophagic pathway promotes the mycobacterial persistence (Figure 2). As previously described (184), we also observed an inhibition of autophagy triggered by live *M. leprae* infection in lepromatous macrophages, however, it can be reverted by IFN-γ treatment. In addition, the levels of autophagy were restored in lepromatous patients who developed the reversal reaction episodes, an inflammatory state associated with increased IFN-γ expression (187). Thus, autophagy is an important innate mechanism associated with leprosy immunopathogenesis.

## Perspectives

The influential role of innate immunity in leprosy biology and their potential as therapeutic targets are now widely recognized. To gain a better understanding of these pathways and to discover new ones, new technologies such as single cell RNA sequencing studies are needed.

Future works should aim to determine further the roles of the neutrophils in host–mycobacteria interaction, with a focus in their role during disease progression. This review supports the role of neutrophils as effector cells and not only as migratory cells following chemoattractants in the context of leprosy. Another promising field that should be investigated by leprologists is the innate lymphoid cell (ILC) biology. ILCs have already been implicated in many studies including metabolism, tissue remodeling and protection against infection.

Although tissue resident macrophages have been extensively studied, phenotypic and functional characteristics of skin resident macrophages and its interaction with the skin sensory nervous system are not fully understood. Furthermore, the dynamic interaction of the resident and the migratory immune cells in the skin may improve our understanding of the immunological events that occur *in situ*. Of note, a recent report demonstrated that nitric oxide secreted by *M. leprae*-carrying macrophages directly damage nerve fibers, by inducing axonal and mitochondrial swelling followed by demyelination phenotype (119). This was a first report showing detrimental roles of infected macrophages that patrols the nerve and induces nerve pathology.

Local Innate immune mechanisms are crucial to determine the outcome of the different clinical forms and the reactional episodes in leprosy patients. The evaluation of the single cell gene expression using RNA sequencing (scRNAseq) emerged as a powerful tool in genomics. scRNAseq provides the expression profile of individual cells. Studies of scRNAseq in leprosy field is a valuable strategy and may shed light on the understanding of the functionality of each cell populations as well as the innate mechanisms induced by *M. leprae* that may contribute for the development of new strategies of control of the disease.

## Author Contributions

Concept of the review: RP and ES. Design and write the review: RP, VS, BJAS, BJS, AD, DE, and MP. Figures and legends: RP, VS, BJAS, MB, and ES. Final approval of the version to be published: RP, VS, and ES.

## Conflict of Interest Statement

The authors declare that the research was conducted in the absence of any commercial or financial relationships that could be construed as a potential conflict of interest.
